# Rapid Posterior Capsular Opacification in Two Patients Treated for Negative Dysphotopsias

**DOI:** 10.21203/rs.3.rs-3907832/v1

**Published:** 2024-01-31

**Authors:** Juan Carlos Navia, Joaquin A. Reategui, Jordan J. Huang, Jaime D. Martinez

## Abstract

**Background:** Negative dysphotopsias (ND) are visual aberrations associated with in-the-bag optic intraocular lens (IOL) placement, causing arc-shaped or linear shadows. Reverse optic capture (ROC) is employed to prevent ND, yet it poses the risk of posterior capsular opacification (PCO) which usually develops within 2-5 years post-surgery due to the lens epithelial cells (LECs) proliferation and migration onto the posterior capsule. This can lead to a cloudy or hazy appearance in the visual field. Early identification of posterior capsular opacities is crucial to ensure timely intervention and minimize visual impairment. **Cases Presentations:** Two cases of acute and rapidly progressive PCO following cataract extraction (CE) and IOL placement using the ROC technique to prevent ND are reported at the Bascom Palmer Eye Institute. At the two-week postoperative follow-up, both patients reported a significant progressive decrease in vision in the treated eye, and severe posterior capsular opacities were observed. A diagnosis of PCO was confirmed, and successful visual rehabilitation was achieved through the performance of ND:YAG laser capsulotomy without complications. This case series represents the first reported instances of patients developing PCO within two weeks of CE and IOL placement using the ROC technique. **Conclusions:**** This case series sheds light on the occurrence of posterior capsular opacities shortly after CE and IOL placement using the ROC technique. It highlights the importance of preoperative patient education, postoperative monitoring, and prompt management of potential complications in cataract surgery.

## Background

Negative dysphotopsias (ND) are visual phenomena described as arc-shaped or linear shadows on the temporal fields of vision appearing mainly with in-the-bag optic intraocular lens (IOL) placement. The etiology of ND is multifactorial; however, the consensus is that light coming from the temporal side passes behind the iris, anterior to the nasal optic edge, without refracting. The shadow that patients see is a result of this "illumination gap".[1, 2]

Different methods have been employed to prevent and treat ND such as piggyback IOL, sulcus haptic placement, and reverse optic capture (ROC), the latter described by Masket and Fram in 2011, which consists of leaving the haptics or loops inside the capsular bag and displacing the optic anteriorly, in order to reduce iris-IOL distance and associated ND.[3] However, despite the success of ROC as a preventive measure against ND, some side effects such as posterior capsular opacities (PCO) have been reported.[1, 4]

PCO itself is the most common complication of cataract surgery. It normally occurs within the first 2–5 years after surgery with a reported incidence between 10–50% after 2 years, with some reports of rapid-onset PCO occurring as early as 3 months post-surgery.[4, 5] IOL material influences the development of PCO with polyacrylic causing the lowest incidence compared to PMMA and silicone lenses due to binding of extracellular matrix proteins to the lens surface.[6, 7] Although PCO can be successfully treated with Neodymium-doped yttrium aluminum garnet (Nd:YAG) laser posterior capsulotomy and surgical posterior capsulotomy, these procedures come with their own risks and costs.

We herein present two cases with acute and rapidly progressive posterior capsule opacities (PCO) after cataract extraction and a silicone intraocular lens placement (CE/IOL) via ROC to prevent ND in the other eye. Reporting this unusual rapid progressive PCO after CE/IOL is important, as it should be considered when discussing possible postoperative complications and managing patient expectations prior to surgery.

### Case Presentation

Two cases (females; ages 70 and 73) with a history of negative dysphotopsias (ND) after cataract extraction and intraocular lens placement (CE/IOL) in one eye underwent the same procedure in the fellow eye due to the presence of cataract. A 3-piece lens with silicone aspheric neutral optics (SofPort LI61AOR2300 Bausch & Lomb Inc.) was inserted using the ROC technique with no intraoperative complications in any of the cases. "There were no previous ocular surgeries on the operated eye, and preoperative manifest refraction was + 1.00 + 0.50@165° (case 1) and + 1.75 diopters sphere (case 2) on the surgical eye." None of the cases had ND after surgery.

In the first case, the corrected distance visual acuity (CDVA) was 20/25 on postoperative day one. Two weeks later the patient presented with a CDVA of 20/100, mentioning the sensation of "*looking through a frosted glass.*" On the slit-lamp examination a posterior capsular opacity (PCO) grade 4 was found (shown in Fig. 1). IOL was centered, with the optic in front of the capsular bag and the haptics inside it, the structures were not rubbing the iris, or any other anterior segment structure confirmed by B-scan ultrasonography. Posterior pole structures were without alterations. Neodymium-doped yttrium aluminum garnet (Nd:YAG) laser capsulotomy was performed without complications and two weeks later the patient presented with a corrected distance visual acuity (CDVA) of 20/20.

In the second case, the corrected distance visual acuity (CDVA) was 20/25 on postoperative day one. Two weeks later her vision decreased to a CDVA of 20/60. On the slit-lamp examination a posterior capsular opacity (PCO) grade 4 was found (shown in Fig. 2), with the IOL centered, with the optic anterior to the capsule and the haptics inside of the bag without rubbing any structure confirmed by B-scan ultrasonography. Posterior pole structures were without alterations. Nd:YAG laser capsulotomy was performed, and two weeks later the patient presented with a corrected distance visual acuity (CDVA) of 20/20.

### Discussion

To the best of our knowledge, this is the first case report describing patients who developed posterior capsular opacification within two weeks of CE/IOL via ROC technique and silicone intraocular lens placement. A study found that primary ROC helped prevent ND in 21/21 eyes of patients who had ND in their first eye and that secondary ROC treated existing ND in 21/22 eyes.[4] In the present study, primary ROC technique was performed in both patients to prevent the development of ND in the fellow eye.

Previous studies by Adre et al. and Masket et al. have described occurrences of PCO after ROC to treat or prevent NDs.[1, 4] However, the former mentions an increased rate of PCO with no time frame, and the latter mentions the incidence of rapid fibrotic PCO and the use of Nd:YAG laser posterior capsulotomy to treat PCO at 3 months in 21 of 21 patients. In both of our cases the PCO occurred in the first two postoperative weeks and were also successfully managed with Nd:YAG laser capsulotomy.

PCO itself, after ROC or other procedures, is a result of surgical injury promoting a wound-healing response. The pathogenesis of PCO begins with trauma to the anterior surface of the lens and disruption of the barrier that holds the Lens Epithelial Cells (LECs) in place. These leftover cells undergo proliferation and slowly migrate towards the posterior lens thanks to cell-cell adhesion molecules. As they settle, they transform into myofibroblasts while the lens capsule undergoes fibrosis, partly due to TGF-B signaling inhibiting LEC proliferation and favoring transdifferentiation.[7]

It has been determined that using a curvilinear continuous capsulorrhexis (CCC) method to open the anterior capsule results in the lowest amount of PCO.[5, 7] However, at the heart of pathogenesis lies the presence of LECs, whose removal is fundamental to PCO prevention since any amount of leftover LECs can lead to a full response. This is evidenced by previous attempts by X. Liu et al. and Shah et al. at polishing the anterior capsule to eliminate LECs which still led to PCO.[7] There is some evidence by Paik et al. that polishing the posterior capsule can cause a reduction in PCO development, albeit this procedure is not widely adapted due to unknown risks.[8] Similarly, it is theorized that removing the posterior capsule through a posterior CCC can permanently eliminate the possibility of PCO, although this too comes with its own risks such as vitreous loss.[7]

Other factors that can influence the development of PCO are the type of material and "wettability" of the IOL. Although there are multiple studies examining the advantages of hydrophobic vs hydrophilic IOLs for PCO development, there is no conclusive evidence to determine which is recommended. When comparing IOL materials, between PMMA, silicone, and acrylic, the latter has shown lower rates of PCO development. In our cases, silicone IOLs were implanted in both patients which could have contributed to the development of PCO. Hollic et al. evaluated 90 eyes after capsulorrhexis and "in-the-bag" IOL placement, and at 2 years all patients with PMMA and silicone had LECs present on the posterior capsule compared to 62% with polyacrylic lenses. Additionally, in those that showed LECs at 90 days, 83% showed regression with polyacrylic compared to 8% with silicone and 15% with PMMA.[6] Similarly, Auffarth et al. described PCO rates of 28.3%, 21.6%, and 8.9% when using PMMA, silicone, and polyacrylic respectively in a group of 1525 patients.[8] These improved results are thought to be because of acrylate IOLs binding extracellular matrix proteins to the IOL surface and promoting capsular adhesion. These proteins bind to LECs and lens capsule, adhere the IOL to the lens capsule and reduce LEC migration.

Another potential factor that can influence the development of a PCO is the location of the IOL haptics and the optic-haptic design. [9] A previous study by Shah et al., on 500 patients undergoing cataract surgery with a 1-piece IOL found a 13.6% incidence of posterior capsular striae. Additionally, the observed rate of PCO development was lower compared to that reported in the literature on 3-piece IOLs. [9] They hypothesize that the IOL haptics play a critical important role in stretching the capsular bag, which prevents the formation of striae and subsequent formation of a PCO. Further, they believe that the optic-haptic design of a 1-piece IOL and the hydrophobic acrylic biomaterial of the haptics, allows for a greater ability to bend backwards, twist, and contort to evenly stretch the capsular bag along its entire circumference. In our study, we used a 3-piece IOL to cover the anterior capsule and positioned the haptics within the ciliary sulcus. This left the posterior lens capsule empty without IOL haptic support and likely caused the capsule to become more susceptable to striae formation, folding, and opacification.

In terms of causes for early opacification, prior vitrectomy, high myopia, indolent rheumatic disease, and post-surgical inflammation have been reported as risk factors. [8–9] In our study, both cases did not have any prior risk factors for early opacification.

In this report, both patients presented 2 weeks post-surgery with reduced CDVA, much faster than reported in previous literature. We believe that PCO developed so quickly in our cases due to a lack of a barrier between the LECs and the lens capsule epithelium, allowing the migration and transdifferentiation of these cells on the posterior capsule. No other reports have shown such rapid progression of PCO and thus warrants attention due to the need for patient education regarding the post-operative possible complications and expectations after cataract surgery.

## Declarations

#### Ethics approval and consent to participate

No ethical approval required.

#### Consent for publication

Written informed consent was obtained from both the patients for publication of the details of their medical case and any accompanying images.

#### Availability of data and materials

All data generated or analyzed during this study are included in this article. Further enquiries can be directed to the corresponding author.

#### Competing interests

The authors have no conflicts of interest to declare.

#### Funding

Supported by NIH Center Core Grant (P30EY014801) (Institutional); Research to Prevent Blindness - Unrestricted Grant (GR004596) (Institutional).

The funders had no role in the design, data collection, data analysis, and reporting of this study.

#### Authors' contributions

Juan C. Navia,  Joaquin A. Reategui and Jordan J. Huang drafted the case report and formatted the manuscript for submission. Jaime D. Martinez-Martinez revised the case report and gave final approval for the version to be submitted.

## Figures

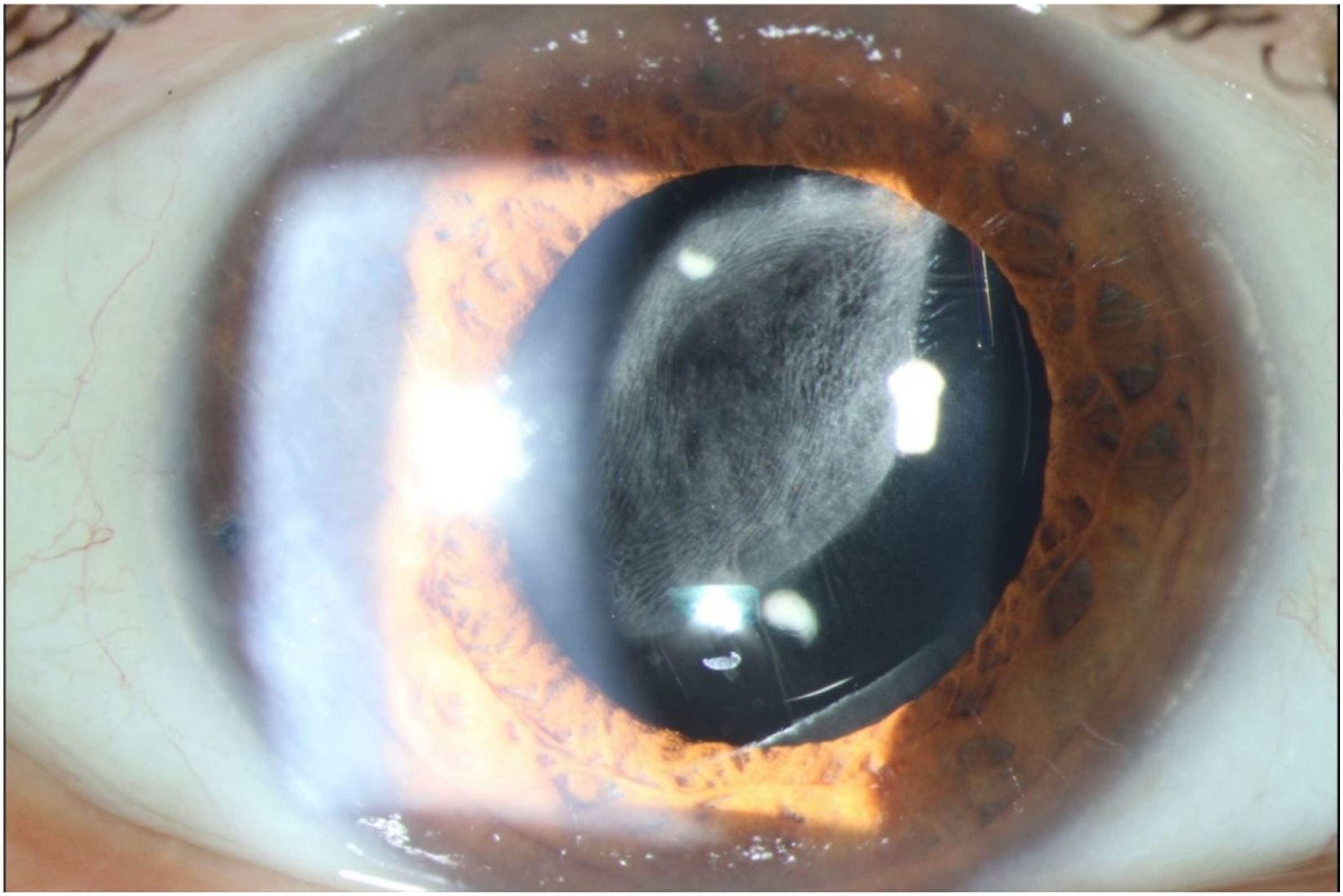

**Figure 1**

Slit-lamp photograph demonstrates posterior capsular opacification 2 weeks after cataract surgery.

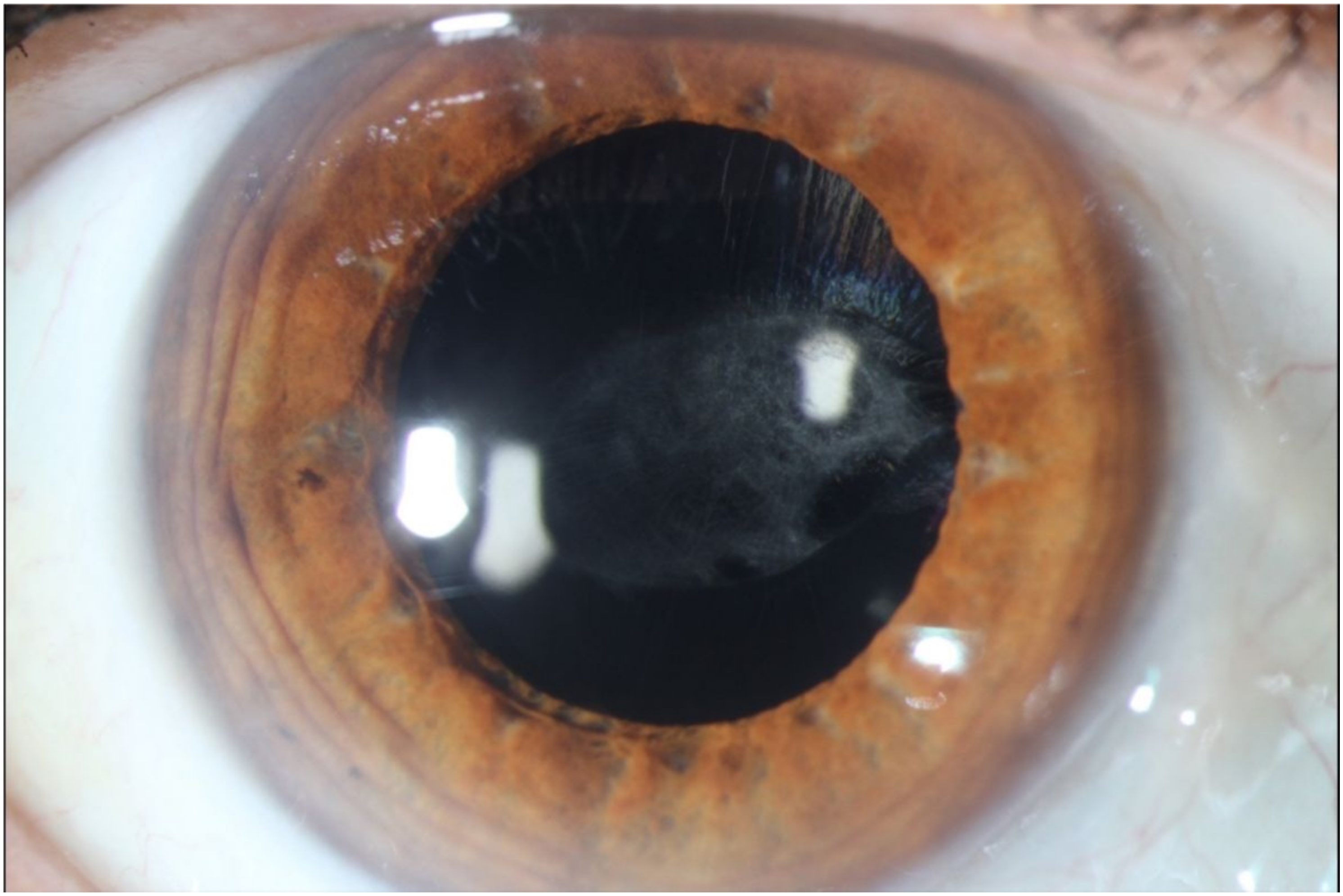

##### Figure 2

Slit-lamp photograph demonstrates posterior capsular opacification 2 weeks after cataract surgery.
